# Plant Protein-Based Delivery Systems: An Emerging Approach for Increasing the Efficacy of Lipophilic Bioactive Compounds

**DOI:** 10.3390/molecules27010060

**Published:** 2021-12-23

**Authors:** Andresa Gomes, Paulo José do Amaral Sobral

**Affiliations:** 1 Department of Food Engineering, Faculty of Animal Science and Food Engineering, University of São Paulo, Pirassununga 13635-900, Brazil; 2 Food Research Center (FoRC), University of São Paulo, Rua do Lago, 250, Semi-Industrial Building, Block C, São Paulo 05508-080, Brazil

**Keywords:** plant-based proteins, functional foods, emulsions, hydrogels, films, delivery systems, encapsulation

## Abstract

The development of plant protein-based delivery systems to protect and control lipophilic bioactive compound delivery (such as vitamins, polyphenols, carotenoids, polyunsaturated fatty acids) has increased interest in food, nutraceutical, and pharmaceutical fields. The quite significant ascension of plant proteins from legumes, oil/edible seeds, nuts, tuber, and cereals is motivated by their eco-friendly, sustainable, and healthy profile compared with other sources. However, many challenges need to be overcome before their widespread use as raw material for carriers. Thus, modification approaches have been used to improve their techno-functionality and address their limitations, aiming to produce a new generation of plant-based carriers (hydrogels, emulsions, self-assembled structures, films). This paper addresses the advantages and challenges of using plant proteins and the effects of modification methods on their nutritional quality, bioactivity, and techno-functionalities. Furthermore, we review the recent progress in designing plant protein-based delivery systems, their main applications as carriers for lipophilic bioactive compounds, and the contribution of protein-bioactive compound interactions to the dynamics and structure of delivery systems. Expressive advances have been made in the plant protein area; however, new extraction/purification technologies and protein sources need to be found Their functional properties must also be deeply studied for the rational development of effective delivery platforms.

## 1. Introduction

In recent decades, interest in consuming lipophilic bioactive compounds has increased, as they are agents that promote health and well-being. A wide range of lipophilic compounds, from plant and animal sources, is considered biologically active, such as vitamins (vitamin D, vitamin E), polyphenols (curcumin, resveratrol, quercetin), carotenoids (β-carotene, lycopene, lutein, α-carotene), and polyunsaturated fatty acids (omega-3) [1]. These compounds can modulate the body’s biological responses, reducing the risk of cancer, cardiometabolic syndromes, cardiovascular diseases, neurodegenerative diseases, and aging-associated diseases [2]. Most of these physiological benefits are associated with bioactive compounds’ antioxidant and anti-inflammatory capacity [3,4]. They have the ability to counteract cellular oxidative stress, which is linked to several metabolism disorders and a series of pathologies [5]. Therefore, the application of physiologically active molecules is an attractive strategy to overcome the challenges of the fast-growing incidence of many chronic diseases around the world.

The health benefits of lipophilic bioactive compounds are often compromised for many different reasons: low water solubility, low membrane permeability, pre-systemic metabolism, low stability in vivo and under environmental conditions. For example, some compounds are unstable in the presence of light, heat, oxygen, and under certain conditions of pH and ionic strength. The poor solubility in water, an intrinsic characteristic of hydrophobic molecules, leads to their insufficient or low bioavailability. Furthermore, many compounds can undergo chemical or biochemical changes in the gastrointestinal tract that influence their metabolic pathway, promoting an additional reduction in their bioavailability [6]. Finally, in addition to low solubility and chemical instability, these molecules have unfavorable organoleptic properties and may undesirably interact with other matrix components, which limits their direct incorporation into functional foods, supplements, and pharmaceutical products [7].

Designing carrier systems is a useful technology for enhancing the oral bioavailability and efficacy of bioactive compounds. Carrier systems can protect the bioactive compounds against adverse external factors, allow their incorporation into water-based matrices, improve their solubility/dispensability in aqueous media, mask unpleasant flavors, allow their controlled release, and preserve their functional properties until they reach the target site [7]. Therefore, the rational selection of material to compose a carrier structure is critical in its development process. Among GRAS materials (generally considered as safe), biomacromolecules, such as proteins, have attracted enormous attention, as they are biodegradable, biocompatible, and can be obtained in abundant quantities from natural renewable sources. In addition, some of them are non-toxic and non-antigenic [8]. These biomacromolecules also meet requirements such as excellent binding capacity with various bioactive molecules, prolonged blood circulation time, and targeting ability [9]. Furthermore, proteins are naturally digested by in vivo enzymes, generating non-toxic metabolites that are readily assimilated by the body [10], and perform biological functions such as muscle maintenance [11], control of immune responses [12], cell signaling [13], and repair of damaged cells [14].

In addition to biochemical and biophysical properties, proteins have versatile functional attributes that make them attractive materials for designing carrier systems. Some of the most important functionalities of proteins include: (i) emulsifying and foaming property—proteins can be adsorbed at water/oil and water/air interfaces, stabilizing colloidal particles [15,16]; (ii) film-forming capacity—the ability to form a cohesive and continuous matrix [17]; (iii) gelling capacity—the ability to form a three-dimensional network [18]; (iv) structure formation—proteins can self-assemble into structures, such as spheres, tubes, or fibers [19,20]; and (v) antioxidant activity—proteins can inhibit oxidative processes through scavenging free radicals and chelation of pro-oxidative transition metals [21]. Different strategies can be used for improving the functionalities of proteins, such as interaction with other compounds (polysaccharides, lipids, emulsifiers, polyphenols) and structural modifications (physical, enzymatic, and chemical) [16,22].

**Figure 1:**
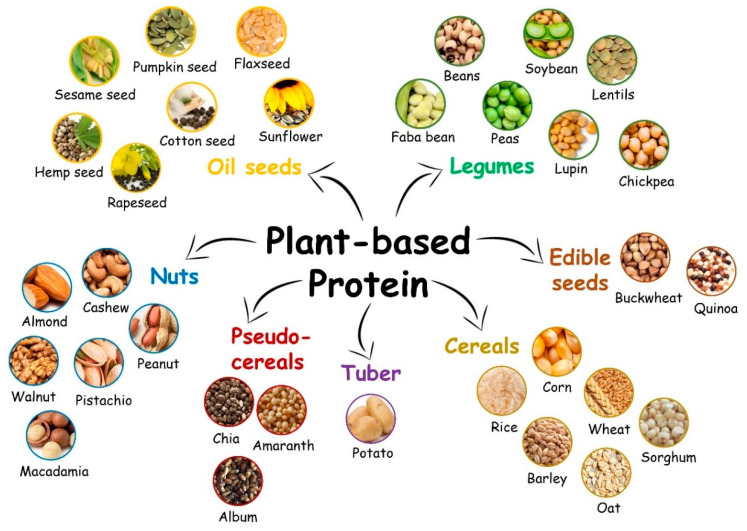
Major sources of plant-based proteins.

Proteins derived from animal sources (meat, fish, eggs, milk) have been broadly exploited to produce carrier structures. Animal-based proteins provide high yield and have excellent functional properties and all the essential amino acids for our body to function efficiently [23,24]. Despite these advantages, there is a fast-growing demand for plant-based proteins as an alternative to their animal counterparts due to new consumer concerns about dietary quality, health benefits, and sustainability [25]. Plant-based proteins can be found in various sources, such as legumes, cereals, nuts, oilseeds, edible seeds, tubers, and pseudo-cereals (Figure 1). In addition to searching for more plant protein alternative sources, extraction methods [26,27,28], modification techniques [22,29], functional properties, and the performance of plant-based proteins as structural building components of the carrier systems [30,31,32,33,34,35] have been investigated recently.

A wide range of carrier structures can be built from plant-based proteins, such as the different types of gels (hydrogel, nanogel, microspheres), solid particles (micro/nanoparticles), micelles, films, protein-based complexes/conjugates, emulsions (micro/nano-emulsion, Pickering emulsion, double emulsion) and more complex structures (emulsion-filled gels and films, micro/nanogel-stabilized Pickering emulsion) [36,37,38,39,40]. However, it is challenging to identify an ideal carrier structure for each lipophilic bioactive compound. The final application of these protein-based carriers depends on the desired release profile, plant-based protein properties, and characteristics of the lipophilic bioactive compounds. Furthermore, interactions between protein and bioactive molecules can modify protein properties and contribute to the structural organization and dynamics of the carrier matrix [41].

Due to the importance of this subject, it has been reviewed recently. For example, Wan, Guo, and Yang [36] reviewed the fabrication of food-grade delivery systems based on plant proteins (zein, soy proteins, wheat gliadins, and barley proteins) using different production methods and their performance in protecting and releasing bioactive food ingredients. Moreover, Malekzad et al. [42] discussed the use of nanotechnology to build drug/gene delivery systems based on plant proteins (zein, gliadin, legumin, lectins). In addition, the authors reviewed the structure and properties of each protein, the different methods of producing carrier structures, and their capacity to target delivery drugs for disorder and disease treatment. Different and complementary, the present review presents state-of-the-art knowledge about utilizing plant-based proteins as raw material for carrier systems for lipophilic bioactive compounds. It addresses the advantages and challenges of using plant-based proteins and the effects of different modification methods on their structure, nutritional value, and techno-functional properties. Furthermore, the different types of plant protein-based carrier systems, their main properties, and the most recent applications in the stabilization, protection, and delivery of lipophilic nutraceuticals will be discussed. Finally, the interaction between proteins and lipophilic bioactive compounds and their contribution to delivery systems’ dynamics and molecular structure will also be explored.

## 2. Advantages and Challenges of Use of Plant-Based Proteins

The plant-based products market is one of the fastest-growing and most innovative sectors of the food industry. The sale of plant-based foods grew by nearly 43% from 2018 to 2020, reaching a market value of around USD 7 billion [43]. Regarding plant-based proteins, the global market has been predicted to reach USD 35.54 billion in 2024, growing at 14% for the period spanning 2020–2024 [44]. There are different reasons for this quite significant ascension. Firstly, plant-based proteins are appreciated by consumers, as they are natural, eco-friendly, and sustainable from the environmental and agricultural points of view [45].

Furthermore, consumers are more knowledgeable and aware about the relationship between a balanced dietary lifestyle, including more plant-based protein sources in their diet pattern, and health claims, such as reducing the risk of cardiovascular disease [46], and reduction of colon cancer migration and inflammation of the lining of the colon (metalloproteinase (MMP)-9 inhibitory activity) [47]. Plant-based proteins also have fewer cultural and religious restrictions and target vegetarians, vegans, and consumers with special dietary needs [48]. Concerning safety, plant-based proteins show a lower risk of infection and contamination than those of animal origin [49]. Furthermore, they are less allergenic and have lower costs than the most used dairy proteins [50,51]. Finally, they are versatile materials, and many consumers still do not accept food-grade proteins from novel sources, such as insects [52,53].

In addition to meeting the increasing consumer demand for natural, renewable, and sustainable products, plant-based proteins have nutritional quality and biological/functional attributes to be a potential alternative to traditional animal-based proteins. These plant-derived biomacromolecules have gelling, thickening, foaming, emulsifying, water retention, and fat absorption properties [54]. They are also an important source of bioactive peptides, which have biological functions of health promotion and disease prevention, including antimicrobial [55,56], antihypertensive [57], antioxidant [58], anticancer [59], anti-adipogenic [60], immunomodulatory [61], and anti-inflammatory [62] effects.

Despite the attractive advantages of plant-derived proteins, many challenges need to be overcome before their widespread use in carrier systems. Indeed, one of the major hindrances to their application is the lack of consistent and desired functional attributes [63]. The native globular structure of plant-based proteins is frequently destroyed by severe conditions present during their extraction and recovery procedures (e.g., high temperature, change of pH/ionic strength, solvent type) [64]. Such processing conditions can cause the denaturation and aggregation of these proteins to different degrees [65], which reduces their water solubility and compromises their main technological properties: (i) properties related to hydration mechanisms (absorption of water/oil, solubility, thickening, wettability); (ii) functional attributes linked to protein structure and rheology (viscosity, elasticity, adhesiveness, aggregation, and gelification), and; (iii) functionalities associated with protein surface activity (emulsifying/foaming activities, formation of protein–lipid films) [64]. For instance, the low solubility in water (less than ~30% at room temperature) influences the behavior of potato, rice, and pea protein commercial concentrates at the oil/water interface and their emulsifying properties. These protein concentrates exhibited restricted interfacial tension, reducing ability, and produced oil-in-water emulsions with a large droplet diameter and limited kinetic stability [66]. A significant number of plant-based proteins are still underutilized because the solubility in water is a prerequisite for their good techno-functional performance in traditional dispersed systems [67,68]. On the other hand, the low solubility of plant-based proteins in both aqueous media and edible oils is now a functional characteristic appreciated in the development of Pickering systems [69].

The complexity and sensitivity to specific environmental conditions can also hinder the application of plant proteins as structure-building materials in delivery systems. For example, during the production steps, the plant-based proteins are submitted to processing conditions (e.g., pressure treatment, high temperature, changes in pH and ionic strength) that can more strongly affect their techno-functionality compared to animal-based proteins. In addition, most plant proteins consist of a mixture of several proteins of different proportions that have different molecular weights, isoelectric points, hydrophobicities, and solubilities, such as flaxseed [70], pea [71], rice, and potato [66]. For example, globulins, which have legumin (11S; 350–400 kDa) and vicilin (7S; 150–180 kDa) as their main fractions, are soluble in dilute saline solution, while albumins (6 to 80 kDa) are water-soluble. As a result of their different amino acid profile, size, and structure, globulins have greater emulsification and gelling capacity than albumins; however, the latter have better nutritional quality and solubility. Within globulins, vicilin has better gelling and emulsifying properties than legumin [72,73]. Furthermore, different extraction procedures may select different proteins, altering the composition of final protein products and their functionalities [28,64]. Lastly, some plant proteins have an undesirable bitter taste that is unacceptable among consumers [74]. Therefore, novel extraction, separation, and fractionation technologies are highly required to modulate the characteristics and improve the functionalities and perception of plant-based proteins.

From a nutritional perspective, plant-based proteins have comparatively lower nutritional value than animal-derived proteins [75]. Plant-source proteins are deficient in one or more essential amino acids, which play a critical role in body maintenance. For instance, legume proteins contain high levels of lysine but are generally deficient in sulfur amino acids (methionine and tryptophan). Contrarily, cereal proteins have higher levels of sulfur amino acids and limited lysine content. Thus, the right combination of proteins, with complementary essential amino acid profiles, can provide a complete and well-balanced amino acid composition [76]. Plant-based proteins are also gastrointestinally less digestible and bioavailable than dairy proteins. For example, proteins such as soy proteins have a highly hydrophobic secondary structure that induces the aggregation process, limiting the accessibility for enzymatic hydrolysis [77]. Furthermore, they contain antinutritional factors, such as trypsin inhibitors, that can further reduce digestion activity and cause low absorption of nutrients [78]. Another challenge is the high allergenicity of some plant-based proteins, such as the ones derived from soy, wheat, and nuts [79].

**Figure 2:**
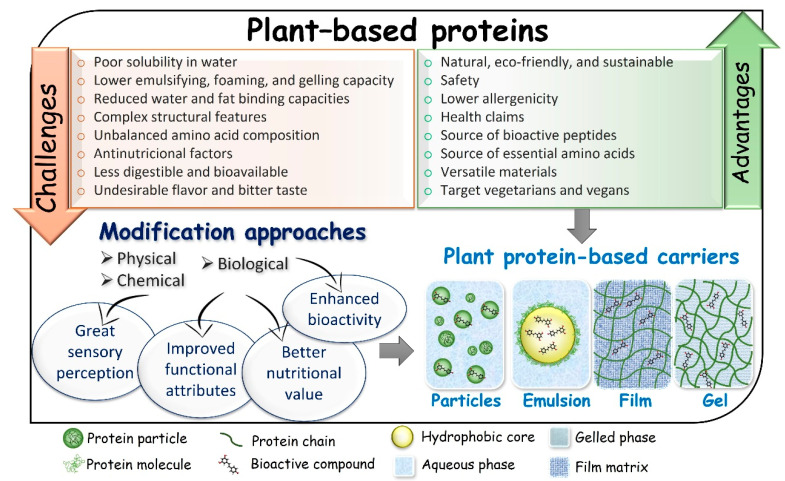
Plant-based proteins: Advantages, challenges, and modification methods aiming at producing different carrier systems for lipophilic bioactive compounds.

To meet market demand for eco-friendly and sustainable products, it is necessary to have plant-based proteins with improved quality and functionality that rival traditional animal proteins. Therefore, the modulation of plant-based protein characteristics to make them more accessible for the production process of carrier systems is highly required (Figure 2).

## 3. Effects of the Different Modification Approaches on Nutritional Quality, Bioactivity, and Techno-Functionalities of Plant-Based Proteins

Plant-based proteins present several challenges that make their application difficult, as shown above. In the face of this, approaches to modifying the physicochemical properties of plant-based proteins have been used to improve their techno-functionality and address their limitations [22]. These shifts are creating new opportunities to innovate and develop multi-functional ingredients to produce a new generation of plant-based products [75,80].

Modification approaches promote perturbations in protein molecules, inducing changes in their thermodynamic state and, therefore, their molecular structure or, specifically, in some of their chemical groups, by altering the molecule’s composition or size and removing or inserting constituents. Modification methods include physical, chemical, and biological processing techniques (traditional and emerging technologies) or a combination of these processing stresses/forces, which can change plant-based proteins’ chemical, biophysical, and surface-active properties. For example, applying some force fields in physical methods can lead to size reduction and redistribution, unfolding, disaggregation, or permanent denaturation of the conformation of proteins [81]. Physical methods comprise techniques such as heat treatment (conventional thermal treatment, ohmic heating, microwave heating, radiofrequency treatment, infrared irradiation), electron beam irradiation, gamma irradiation, ultraviolet radiation, pulsed-electric field, high-pressure treatment, sonication, extrusion, ball mill treatment, cold atmospheric plasma processing, or ultrafiltration [22]. Differently, chemical modification of the proteins is carried out by adding new functional groups or removing components from their structure, employing techniques such as glycation, phosphorylation, acylation, deamidation, cationization, or pH shifting treatment. However, despite having improved functional properties, the application of chemically modified proteins encounters resistance due to the harmful consequences of possible chemical residues [81]. On the other hand, biological methods are environmentally friendly, do not lead to toxic by-products, and preserve the protein’s initial chemical composition. Furthermore, they can improve the proteins’ nutritional quality and antioxidant/antimicrobial properties through enzymatic and fermentation processes. Another attractive modification method is complexation, which is based on the chemical diversity of plant proteins and their binding capacity with different compounds. Furthermore, plant-based proteins can interact with other proteins or compounds (polysaccharides, lipids, amino acids, emulsifiers, polyphenols), designing novel bioparticles with tunable properties [22].

The selection of an appropriate modification method should consider the functionality of interest and the final application of plant-based proteins. Proteins are complex biomacromolecules, and their rational modification requires an in-depth knowledge of their nature and structural properties and the mechanism needed to reach a modified target functional property. In this sense, it is essential to emphasize that several attributes of proteins can be affected by applying only one modification technique or by a combination of different techniques. Table 1 presents recent studies on tailoring plant-based protein properties by protein modification techniques. The effects of different modification strategies on the nutritional value, bioactivity, sensory attributes, and techno-functionality of plant-based proteins are discussed in more detail below.

### 3.1. Effects on Properties Related to Hydration Mechanisms

One of a protein’s most critical functional characteristics is its water solubility, as this attribute can narrow various other functionalities. Plant-based proteins, in general, exhibit low solubility in water, and this limitation has been a hindrance to their broad application. Therefore, the modulation of plant proteins to increase their solubility and improve their functionalities is highly sought. Several traditional or innovative modification techniques have been employed to face these challenges, and notable advances have been made in this area. Pulsed-electric field technology was applied in wheat gluten proteins (82.4% protein), and an improvement in their solubility, water holding capacity, and oil holding capacity was observed with increasing electric field intensity (0–12.5 kV cm^−1^) [100]. Greater electric field intensities can cause a great unfolding and extension of the protein molecules, which expose the hydrophilic groups inside the molecules, increasing the absorption of water and strengthening the interaction between water and protein molecules. Furthermore, proteins expose non-polar aromatic and aliphatic amino acids on the surface during unfolding, thereby increasing the surface hydrophobicity and the ratio of hydrophobic to hydrophilic amino acids influencing the oil holding capacity [101]. However, in some intensity and time residence conditions, applying the pulsed-electric field causes denaturation and aggregation of proteins, worsening their interaction with water and oil [100]. Likewise, the solubility and surface hydrophobicity of ultrasound-treated album (*Chenopodium album)* protein isolates (43 W/cm^2^, 500 W, 0–35 min) gradually increased with increasing process time up to 25 min (solubility, from 70% to 94%; hydrophobicity, from 124 to 167), while a significant decrease was observed after sonication for 35 min (92%; 160) [102]. The ultrasound treatment (0, 150, 300, 450, and 600 W for 15 min) also enhanced the solubility and dispersibility in the water of the soy protein isolate-pectin complexes. The solubility increased from 46.42% (untreated sample) to 89.88% after sonication at 600 W [103]. The cavitation generated during sonication can destroy the non-covalent bonds that maintain proteins’ spatial structure, promoting their unfolding and reducing the complex size and, thereby, improving the complex solubility and dispersion in an aqueous solution [103]. On the other hand, dry heating at 75 and 100 °C promoted a gradual reduction in the solubility of the bean protein concentrate with increasing temperature [104].

In addition to the physical methods mentioned above, the interactions of plant proteins with other molecules, such as other proteins, amino acids, and polysaccharides, can tune their properties associated with hydration mechanisms. For example, the complexation with whey protein isolate (rice protein/whey protein ratio of 1:1) boosted the solubility of rice proteins from 1.7% to over 50% [105]. A further increase in the solubility of rice protein, more than 40-fold, to 82%, was obtained after its complexation with soy protein isolates at a rice protein/soy protein isolates ratio of 1:0.1 [106]. Furthermore, a recent study reported that the interaction between zein and glutamic acid in different zein/glutamic acid ratios (1:0, 1:0.01, 1:0.05, 1:0.1, 1:0.2, 1:0.3, 1:0.4) affected the zein wettability. The oil contact angle of zein/glutamate particles increased continuously, decreasing the zein/glutamic acid ratio down to 1:0.01 (from 46.24 to 84°), indicating an increase in zein wettability in the oil phase [107]. The addition of sodium caseinate to zein, at zein/sodium caseinate ratios (*w*/*w*) ranging from 10:1 to 10:4, also enhanced the zein wettability in the oil, and intermediate wettability was achieved at zein/sodium caseinate ratio of 10:4 (85°) [108]. A similar improvement in zein wettability (78.30°) was obtained when the surface of zein particles was modified with pectin (0.05% *w*/*v*) [109].

### 3.2. Effects on Functional Attributes Linked to Structure and Rheology

Proteins play a crucial role in forming structure and imparting texture, as they can form aggregates and gels. Under certain environmental conditions, proteins become more flexible, facilitating the exposition of free sulphydryl groups, which can form disulfide bonds with free sulphydryl groups on neighboring proteins, forming aggregates. The association between protein primary aggregates through sulphydryl/disulfide reactions propagates the aggregation process, forming a three-dimensional network able to entrap water (if the protein concentration is high enough), thus forming a gel. Other mechanisms, such as hydrogen bonds, hydrophobic, van der Waals interactions, can also stabilize the protein–protein interactions [110]. The aggregation and gelation of proteins are influenced by their characteristics, such as hydrophobicity, hydrophilicity, charge distribution, conformation, size, and amino acid composition [111]. In this sense, based on the relationship between the structure and functional properties, the application of modification processes capable of altering protein molecules’ structure, conformation, and size can be a helpful tool to adjust their rheological and gelling properties. These approaches have been extensively used to modify plant-based proteins, since some have shown restricted structure building capacity. For instance, the application of ultraviolet radiation (0.8 mWm^−2^, 32.6 Jm^−2^, 6 h) in a film-forming solution based on sesame protein isolate resulted in a crosslinked film with improved mechanical properties. The radiation provoked an increase in ultimate tensile strength (from 5.14 to 8.29 MPa) and Young’s modulus (from 68.15 to 118.35 MPa), and a reduction of elongation at break (from 105.38 to 65.29%) [112]. Differently, films produced by chemically modified potato protein (heating at a basic pH) at 130 °C showed a lower Young modulus value and higher extensibility [113].

Controlled heat treatment (80–100 °C for 15 or 30 min) also modified the thermal and gelling properties of album (*Chenopodium album*) protein isolate. Heat-treated proteins showed lower denaturation enthalpy and higher denaturation temperature, measured by differential scanning calorimetry, and loss and storage moduli, determined by dynamic rheology, than the native protein [114]. Similar improvement in chickpea proteins’ viscoelastic properties (higher storage and loss of moduli values) was obtained after high-intensity ultrasound treatment (300 W for 5, 10, or 20 min). Moreover, the sonication reduced the viscosity of chickpea protein solutions, and the gels produced with these sonicated solutions showed a higher breaking force (150–201.4 g) than that untreated gel (78.1 g) [115].

Another non-thermal modification technique, the cold atmospheric plasma, has gained attention as a potential alternative to traditional thermal treatment. A recent study reported that cold atmospheric plasma treatment reduced the denaturation temperature of pea protein (70–90 °C), promoting an improvement in its gelling properties. The native pea protein suspension (12 wt%) could not form a gel after heating at 100–120 °C for 30 min, and a fragile gel was formed (95 °C for 30 min) after increasing protein content (15 wt%). On the other hand, pea protein suspension treated by cold atmospheric plasma formed strong and elastic gels by heating at 80–90 °C. Furthermore, the gel formed at 80 °C from treated pea protein (12 wt%) showed higher mechanical strength (2.70 kPa) than that of native pea protein gel (15 wt%) heated at 95 °C for 30 min (1.03 kPa) [116]. Atmospheric cold plasma induced protein unfolding at reduced temperature, contributing to the formation of protein fibrillar aggregates. Furthermore, this technique increased the exposition of free sulphydryl groups and the content of disulfide bonds in plant-based proteins, resulting in changes in their secondary structure, which led to gels with improved mechanical properties [116].

### 3.3. Effects on Functionalities Associated with Protein Surface Activity

The proteins, due to their amphiphilic nature, can adsorb at the oil/water or air/water interfaces and promote a reduction in the tension between the phases, efficiently stabilizing multiphase systems, such as emulsions and foams. At the interface, proteins form a viscoelastic layer around the droplets and bubbles, preventing the destabilization of the emulsions and foams through electrostatic repulsion and steric hindrance. The emulsifying and foaming properties of proteins are amongst their most important functional properties. Such attributes are affected by protein characteristics, such as molecular weight, flexibility, water-solubility, the distribution pattern of hydrophobic and hydrophilic groups, and the surface hydrophobicity/hydrophilicity ratio [117]. Recently, several studies have reported that plant-based proteins possess promising emulsifying and foaming properties, which can be modulated through modification techniques [118]. For instance, the emulsifying properties of Great Northern and navy bean (*Phaseolus vulgaris* L.) protein concentrates were enhanced by heat treatment-assisted enzymatic hydrolysis. Emulsions stabilized by alcalase hydrolysates (enzyme/protein ratio of 80:1000) and papain hydrolysates (enzyme/protein ratio of 5:1000) showed smaller droplet sizes and better stabilities than those produced with untreated proteins from either source [119]. Similarly, emulsions stabilized by pea protein gum Arabic conjugates, produced by controlled Maillard reaction for 1 or 3-day incubation, showed smaller droplet diameters and greater physical stabilities against pH, temperature, and ionic strength than pea protein-stabilized emulsion. However, extending the incubation time decreased the emulsifying capacity, and emulsion stabilized by 5 day-incubated conjugates showed higher droplet size and trimodal size distribution curve [120].

The microbial transglutaminase treatment for 120 and 240 min also decreased the emulsifying activity of faba bean proteins by 17% and 39%, respectively, due to excessive surface hydrophobicity [121]. On the other hand, transglutaminase treatment improved the emulsifying and foaming properties of peanut protein hydrolysates [122]. Hydrolysis of peanut (*Arachis hypogea* L.) protein concentrate by fungal crude protease extract also improved in its foaming capacity and foaming properties [123]. The highest foaming capacity value of 58.70% was observed in samples hydrolyzed with protease obtained from *Aspergillus oryzae* followed by *Rhizopus oligosporus* (47.31%) and *Trichoderma reesei* (33.66%). In comparison, the peanut protein concentrate showed a foaming capacity value of 22.33%. Similar behavior could be observed concerning foaming stability (*A. oryzae* > *R. oligosporus* > *T. reesei* > peanut protein concentrate). The enzymatic hydrolysis treatment led to smaller particle size and greater solubility, which improved the protein’s surface properties and, therefore, its foaming ability and foam stability [123].

### 3.4. Improvement of Perceived Sensory Acceptance

The bitter sensation of plant-based proteins and their derivatives is mainly promoted by electrostatic and hydrophobic forces between the bitter substances and the taste receptors in the mouth. An alternative to masking bitterness is the complexation of these proteins with polysaccharides. For example, the complex coacervation of pea and potato proteins with apple pectin reduced the bitter off-notes in sensory evaluation with increasing pectin/protein ratios [74]. Regarding flavor attributes, the conjugation of pea protein concentrates with gum Arabic by controlled Maillard reaction (60 °C and 79% relative humidity) mitigated the beany flavors. The content of the bean flavor markers was below 1 ppm after the 1-day incubation, and some of them disappeared after 5 days of incubation [120]. In protein hydrolysates, releasing peptides containing hydrophobic amino acid residues can intensify the bitter taste. Zhang, et al. [124] reported that the combined use of controlled alcalase hydrolysis and transglutaminase crosslinking decreased the bitterness and improved the overall flavor of soybean protein hydrolysates. The bitterness of hydrolysates can also be addressed by other methods, such as deamidation-induced modification. For example, wheat gluten hydrolysates produced by deamidation-enzymatic hydrolysis showed an enhanced bitterness-masking effect, attributed to an increase in umami flavor amino acids [125,126].

### 3.5. Enhancement of Nutritional Value

Proteins are fundamental macronutrients in human nutrition and health. However, some plant-based proteins can cause allergic reactions and contain various anti-nutritional compounds (e.g., trypsin inhibitors, tannins, phytic acid, and α-galactosides) that induce undesirable physiological effects. Various methods of structural modification have been employed to overcome these plant-based proteins’ limitations, including gamma irradiation [127], ultrasound [128], electron beam irradiation [129], high pressure [130], or a combination of techniques such as enzymatic hydrolysis followed by transglutaminase crosslinking [122]. For instance, thermally treated album proteins (100 °C for 30 min) showed enhanced digestibility (87.55%) and availability of their essential amino acids. Heat treatment changed album protein conformation and created additional binding sites for the proteolytic enzymes [114]. Likewise, ultrasound and microwave treatment significantly reduced trypsin inhibitor activity and improved the protein digestibility in soymilk. Digestibility of soymilk was 15.68% higher after microwave processing (85 °C for 10 min), while ultrasound treatment (16 min) reduced trypsin inhibitor activity by 52% [78].

Anti-nutritional factors can also be reduced by biochemical conversion through fermentation processes. The fermentation with a lyophilized yogurt culture reduced by 2.7% the trypsin inhibitor activity in chickpea flour and simultaneously increased its in-vitro digestibility by 9.5% [131]. A solid-state fermentation with *Pediococcus* spp. also decreases the amounts of indigestible α-galactosides (90%) and phytic acid (17%) in chickpea protein concentrate [132]. In addition, fermentation can affect the allergenicity of plant-based proteins, depending on the starter culture type. For example, *Lactobacillus helveticus*-fermented soy protein isolate showed up to 100% reduction in immunoglobulin E reactivity, while the fermentation induced by *Lactobacillus plantarum* strains decreased by 83.8–94.8% [133]. Chemical modification techniques through deamidation by cation-exchange resins could also decrease the oral allergenicity in vitro and in vivo of gliadin, a major allergen in wheat [134].

### 3.6. Positive Effects on the Biological Functions

In recent years, an emerging interest has been to improve the bioactivity of plant-based proteins and their derivatives through structural modification techniques. Relevant advances have been observed in this field. For instance, the pretreatment of wheat germ protein with electron beam irradiation (0–50 kGy) promoted a sharp rise in the antioxidant activity of their hydrolysates. The scavenging activities of 1,10-diphenyl-2-picrylhydrazyl (DPPH) and 2,2-azino-bis(3-ethylbenzthiazoline)-6-sulfonic acid (ABTS) of the pre-irradiated hydrolysates at 50 kGy increased by 45.77% and 52.52%, respectively, compared to the non-irradiated sample. [135]. In addition to having an increased DPPH (32.06%) and ABTS (79.11%) radical scavenging capacity, electron beam irradiation-pretreated rice protein hydrolysates (30 kGy) showed marked improvement in cellular antioxidant activity values (51.19%) [136]. In a more complex modification process of the linseed protein, based on the simultaneous pressurization (300 MPa for 10 min) and trypsin hydrolysis, the antioxidant activity in terms of oxygen radical absorbance capacity (ORAC) of the hydrolysates was 35% greater than control at atmospheric pressure [137]. The combination of modification techniques, such as microwave-assisted hydrolysis, also improved the antidiabetic activity of chia seed (*Salvia hispanica*) proteins, although no change in their antihypertensive activity was observed [138]. The energy generated in some processes, such as irradiation, high pressure, and microwave, can induce unfolding and/or scission of protein molecules, exposing more peptide bonds to enzyme hydrolysis. These changes allow the protein breakdown in smaller molecular weight peptides with higher bioactivity [139]. The modulation of bioactivity plant-based proteins can also be reached by other methods, such as fibrillation and complexation with phenolic acids. For example, the antioxidant activities of soy protein fibrils (50%) were higher than those of soy proteins (20%), and a further increase in the ABTS radical scavenging activity was observed in ultrasound-treated soy protein fibrils (58%) [140]. The antioxidant capacity of fibrils was once more increased after their complexation with Epigallocatechin gallate, and antioxidant activity of ultrasound-treated fibrils complexed with Epigallocatechin gallate was 90% [140]. Ultrasonication and the fibrillation process promoted modifications in protein molecules, resulting in greater exposure of their hydrophobic groups, hydrophobic amino acid residues, and peptides and amino acids production. Such processes exposed more active protein/fibril sites to interact with epigallocatechin gallate, resulting in even greater antioxidant activity [140].

The modification techniques, as shown previously, are a helpful tool for overcoming the limitations of plant-based proteins, which limit their application as raw material to build delivery platforms for lipophilic bioactive compounds. These approaches can modify protein structures and their physicochemical properties and, therefore, change their capacity of self-assembly, emulsion/foam stabilization, gel and film formation, and their bioactivity, nutritional value, and sensory acceptance. There are various modification methods capable of altering a specific property or a group of them, and the combination of techniques can be an attractive strategy to obtain improved results. However, it is essential to highlight that the processing conditions (time, temperature, enzyme type, pressure, crosslinking agent, concentration, energy density) used within a method can positively or negatively affect plant-based proteins’ functionalities. Therefore, the modification technique and conditions of the process should be previously tested for each protein, so that the modified plant-based proteins can become competitive materials to partially or fully replace animal-based proteins.

## 4. Designing Plant Protein-Based Carriers

**Figure 3:**
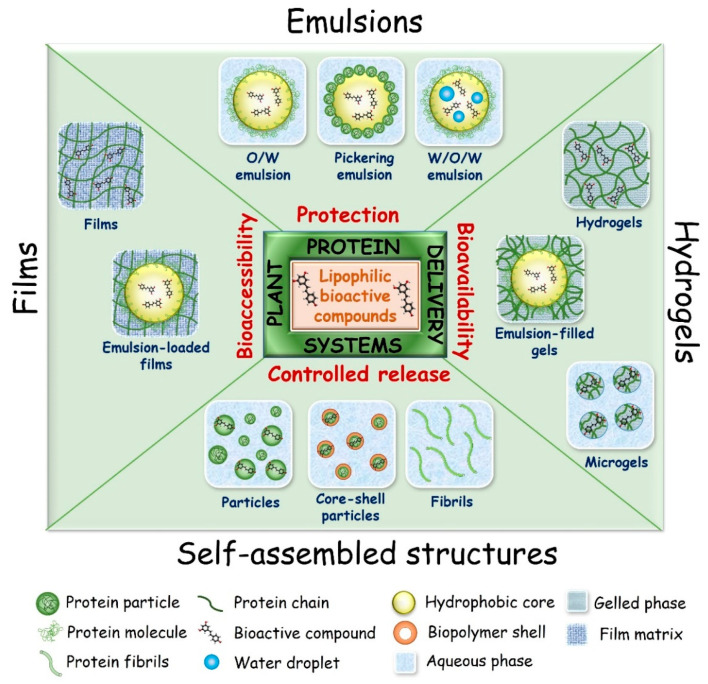
Overview of the different plant protein-based delivery platforms.

It is well known that plant-based proteins are biomacromolecules with diversified functional properties, including emulsification, gelation, self-assembly, film-forming ability, and even intrinsic nutraceutical activity [47]. Their chemical and structural versatility makes them appropriate as raw material for soft-carrier materials to build a wide range of platforms (such as particles, fibers, films, gels (hydrogel, nanogel, microspheres), micelles, complexes/conjugates, emulsions (micro/nano-emulsion, Pickering emulsion, double emulsion) and multifaceted structures (emulsion-filled gels and films, micro/nanogel-stabilized Pickering emulsion, complex/conjugate-stabilized emulsion)), which can encapsulate, retain, protect, and deliver hydrophobic bioactive compounds (Figure 3). However, it must be emphasized that there is no ideal carrier platform for a lipophilic bioactive compound. In addition to specific advantages and disadvantages of each carrier system, the protein type (source and structural shape), bioactive compound characteristics (solubility, surface properties, hydrophobicity, molecular weight), biological action, target site, and release profile of bioactive compound must be considered for the successful development of delivery systems [141,142,143]. Different strategies have been used in designing delivery systems, and notable advances have been made in the protection and bioaccessibility/bioavailability of bioactive compounds, as shown below. Recent studies on encapsulation of lipophilic bioactive compounds using plant-based proteins as carrier material are presented in Table 2.

### 4.1. Self-Assembly Structures

Proteins or peptides can self-assemble into versatile architectures with different symmetries and sizes (e.g., nanofibers, nanotubes, particles, micelles, fibrils, and complexes) [160]. The self-assembly of proteins involves various supramolecular interactions, such as hydrophobic interactions, amphiphilic interfaces, hydrogen-bond networks, and Van der Waals interactions. These interactions are triggered by different physicochemical strategies and approaches, including heating, enzymatic hydrolysis, pH-shifting, urea or ethanol treatment, reduction, and static high-pressure treatment [20,161]. The self-assembled structures exhibit dynamic behavior, novel mechanical attributes, and great potential to act as carrier platforms for lipophilic bioactive compounds. The protective effect and delivery ability of assembled architectures based on plant proteins have been demonstrated by many studies. For example, curcumin was effectively encapsulated (97.43%) in the reassembled soy protein nanoparticles formed by the pH-shift method. The encapsulation significantly improved curcumin’s water solubility, thermal stability, and photostability [162]. Furthermore, soy protein nanoparticles self-assembled via partial enzymatic hydrolysis, protected the curcumin during simulated gastric-intestinal digestion, and remarkably increased its gastrointestinal bioaccessibility (80%) compared to free curcumin (10%). The curcumin-loaded soy protein nanoparticles also attenuated H_2_O_2_-induced oxidative damage on HepG2 cells, ensuring high cell viability (81.30%) [163].

In addition to self-assembling of protein alone, plant-based proteins can co-assemble with other biopolymers to form more stable architectures with distinct properties. The surface modification of zein particles or the complexation of zein with other proteins and polysaccharides, such as whey protein [164] and ι-carrageenan [165], improved the stability of zein particles and their ability to protect lipophilic bioactive compounds. The combination of zein, propylene glycol alginate, and rhamnolipid also formed composite nanoparticles with improved physicochemical stability, promoting sustained resveratrol release in the small intestine [166]. Furthermore, core-shell nanoparticles, produced using zein-epigallocatechin gallate conjugates as the core and the rhamnolipids as the shell, showed high loading efficiency of curcumin (71%) and resveratrol (85%). The encapsulation in the core-shell nanoparticles protected these nutraceuticals from degradation, preserving their antioxidant activity; however, the bioaccessibility of curcumin (27.9%) and resveratrol (42.7%) was relatively low [167]. On the other hand, the alginate/sodium caseinate-coated zein nanoparticles prolonged curcumin release under simulated gastrointestinal tract conditions and increased its bioaccessibility (69.1–95.5%) compared to free curcumin (47%) [168]. A similar result was obtained in a recent study in which nanoparticles of rice protein modified with carboxymethylcellulose were used as a lutein delivery system [169]. Coating the rice protein particles with carboxymethylcellulose delayed the lutein release in the stomach (8.10%) and gradually increased its release in the small intestine step after 6 h of digestion (96.07%). In addition, lutein-loaded rice protein-carboxymethylcellulose nanoparticles inhibited the proliferation of breast cancer cells (MCF-7) with an inhibition rate of 70% in a dose-dependent manner when lutein concentration was 20 μM [169].

### 4.2. Emulsions

Emulsions are colloidal dispersions containing two immiscible phases, commonly oily and aqueous, in which one phase is dispersed into another as droplets. They can be oil-in-water or water-in-oil conventional systems or more sophisticated dispersions (Pickering emulsions and double/multiple emulsions). A kinetically stable emulsion is obtained by adding emulsifiers, such as plant-based proteins, which form an interfacial layer around the droplets, preventing destabilization by gravitational separation (creaming) or droplet aggregation (flocculation, coalescence, and Ostwald ripening) [170]. The emulsions stabilized by plant-based proteins can protect, carrier, and target delivery lipophilic bioactive compounds vehiculated within the hydrophobic core of their lipid droplets. According to McClements [171], these systems can improve the functional compounds’ water solubility and chemical stability, ensuring their improved bioaccessibility, bioavailability, and efficacy. For instance, encapsulation of quercetin in nanoemulsions stabilized by rice bran protein improved its bioavailability (12.70%) and cell permeability (4.93 × 10^−6^ cm/s) compared to free quercetin (1.40% and 2.9 × 10^−6^ cm/s). This system also demonstrated excellent encapsulation efficiency (98.12%) and stability under alkaline conditions and low salt ion concentrations [172]. Similarly, pea protein-stabilized nanoemulsions showed good kinetic stability and high vitamin D encapsulation efficiency (94–96%). The encapsulation of vitamin D in nanoemulsions enhanced its uptake in Caco-2 cells by 5.3-fold compared to the free vitamin D suspension [173]. Furthermore, the consumption of vitamin D vehiculated in nanoemulsions stabilized by ultrasound and pH-shifting-treated pea protein improved the vitamin D serum levels in vitamin-deficient rats [174]. A further increase in bioaccessibility was reached when the quercetin was vehiculated in water-in-oil-in-water (W/O/W) emulsions (58.7%) compared to the oil-in-water (O/W) system (52.33%). In addition, the black bean protein-stabilized W/O/W double emulsion had a high encapsulation efficiency (93.4%) and controlled the release of quercetin during digestion. The antioxidant activity of black bean protein was able to well inhibit the lipid oxidation, with low levels of hydroperoxides (0.86 mmol/L) and TBARS (25.80 μmol/L) [175].

The interaction of plant-based proteins with other proteins or polysaccharides can tune their emulsifying properties and their performance as carrier materials in Pickering emulsified systems. For example, the stabilization of Pickering emulsion with wheat gluten-xanthan gum complex improved the β-carotene chemical stability during storage, with around 94.3% of carotenoids retained after one-month storage at 25 °C [176]. Furthermore, the addition of xanthan gum reduced the aggregation of the oil droplets in the gastrointestinal fluids, facilitating the lipase access to the surface of corn oil droplets and increasing the amount of β-carotene solubilized in the mixed micelles by around 18% [176]. The conjugation of *Pleurotus eryngii* polysaccharide with soy protein via Maillard reaction also facilitated the lipid digestion of Pickering emulsion, leading to an increase in the β-carotene bioavailability. In addition, the gastrointestinal behavior of this system ensured higher content of β-carotene in an active state for relieving oxidative stress in Caco-2 cells [177].

The complexation of plant-based proteins with polysaccharides via electrostatic attraction has also been used to fabricate more complex delivery systems, such as Pickering high internal phase emulsions (HIPEs). For instance, the stabilization of HIPEs with pea protein-high methoxyl pectin colloidal particles resulted in highly stable systems, regardless of pH variation, which effectively protected the β-carotene against thermal degradation at 50 °C for 30 days of storage (retention rate of 58–70%) [178]. Likewise, the pecan protein-xanthan gum complex interfacial film contributed to the high retention of quercetin in Pickering HIPEs when exposed to heat, iron ions, and hydrogen peroxide in the aqueous phase. Furthermore, the bioaccessibility of quercetin in Pickering HIPE (40.0%) was significantly higher than that dispersed in oil (29.9%) [179].

### 4.3. Hydrogels

Hydrogels are produced by forming junctions of polymer chains (such as plant-based proteins), which leads to the formation of a three-dimensional network with high water holding capacity. They have a semi-solid intermediate structure that retains both liquid and solid-type rheological behaviors. Thus, hydrogels have a great potential for application, as they can be designed to adapt textural properties, in addition to being platforms for carrying bioactive compounds [180]. The biopolymeric network can protect the hydrophobic bioactive ingredients against adverse environmental conditions, such as oxygen, heat, light, acids, and enzymes, preventing their degradation during storage and in the stomach. Furthermore, the hydrogel structure allows the release of functional molecules in the small intestine, enhancing their uptake and utilization. For instance, pinto bean protein-based acid-induced cold-set gels presented an excellent embedding potential of curcumin (98.54%). The gelled structure also delayed curcumin’s release in a simulated gastric medium (22.6% after 2 h) and led to its sustained release in a simulated intestinal condition (61.68% after 4 h) [181]. The cold-set NaCl-induced gelation of the continuous phase of the emulsion also prevented the degradation of β-carotene encapsulated in solid lipid particle-filled gel composed of soy protein and locust bean gum. After 30 days of storage, the emulsion-filled gel retained about 90% of the encapsulated β-carotene, while solid lipid particles retained only 57% [182]. In addition to gelation, the type of coagulant (CaCl_2_, GDL, and transglutaminase—MTGase) and homogenization process (ultrasound or mechanical) modulated the β-carotene bioaccessibility encapsulated in emulsion-filled gels based on soy protein. Both gelation of the continuous phase and ultrasound treatment facilitated the lipid hydrolysis and solubilization of the β-carotene in mixed micelles. Regarding the type of coagulant, MTGase-induced bulk emulsion gels exhibited higher bioaccessibility of β-carotene (around 83%), which was related to its higher gel strength [183]. Similarly, the chemical stability and bioaccessibility of β-carotene in emulsion-filled gels based on soy protein-pectin composite improved after high-intensity ultrasound treatment (0, 150, 300, 450, and 600 W). The cavitation caused by high-intensity ultrasound reduced the oil droplet size and the particle size of soy protein and pectin, which increased the protein-polysaccharide interactions and the stability of the network structure. Such structure decreased the aggregation of oil droplets during digestion and slowed down the diffusion of prooxidant substances to the droplet surface, which improved lipid digestion and delayed the β-carotene degradation.

The chemical stability (78.3%) and bioaccessibility (83%) of β-carotene after in-vitro digestion, when treated with high-intensity ultrasound at 450 W, were higher than those of the emulsion gel without ultrasound treatment (54.8%; 65%, respectively) [184]. Blends of plant-based proteins and polysaccharides were also used to build fibers via electrospinning technique to encapsulate quercetin. For example, fibers composed of amaranth protein-pullulan blend allowed the sustained release of quercetin during in-vitro digestion, contributing to the improvement of its antioxidant capacity compared to the free quercetin [185].

### 4.4. Films

In recent years, active films based on biopolymers, such as plant-based proteins, have gained increasing attention due to their biodegradable, renewable, and environmentally friendly characteristics [186]. They are formed by incorporating bioactive compounds inside the film biopolymeric matrices, promoting their controlled delivery. In addition to the traditional packaging functions, active films can have different roles, such as oxygen and ethylene scavenging, carbon dioxide emitting, antimicrobial and antioxidant protection, and others, depending on the incorporated bioactive compounds into films [187]. Lipophilic active compounds can be directly added to the film or previously loaded in other systems, including particle encapsulation, oil-in-water micro/nanoemulsions, water-in-oil-in-water emulsions, Pickering emulsions, solid-lipid micro/nanoparticles, multilayer system, and electrospinning technology. These structures can increase the dispersibility of lipid-soluble compounds into the water-solubilized biopolymer matrix, protecting them against degradation and improving their retention and sustained release, promoting an enhancement in their bioactivity [188].

Soy protein-based films incorporated with oregano essential oil-loaded microparticles or with the addition of free oregano essential oil demonstrated the formation of an inhibition halo for both *Escherichia coli* and *Staphylococcus aureus*. However, the inhibition halo of the film with oregano essential oil-loaded microparticles was significantly higher for both bacteria than that with the addition of free oregano essential oil [189]. Soy protein-based films loaded with carvacrol also exhibited antimicrobial activity against *Listeria grayi* and *E. coli* K12 in direct contact with the bacteria. In addition, the carvacrol vapor released from these films was also effective in inhibiting the growth of *L. grayi* [190].

The diffusion rate of active compounds through the films can be modulated by the addition of nanocomposites, which affect the biopolymeric network characteristics and film tortuosity. For instance, soy protein-based films nano-reinforced with montmorillonite-MMT (10 g MMT/100 g soy protein) and activated with clove essential oil decreased the microbial growth and lipid autooxidation of tuna fillets for 17 days of storage at 2 °C. The presence of montmorillonite favored the release of the active principles of clove oil by prolonging its antimicrobial and antioxidant activity over time, especially effective in inhibiting *Pseudomonas* spp. [191]. Likewise, the addition of micro-fibrillated cellulose favored the release of the active compounds of clove essential oil in soy protein-based films, probably because the nanofibers improved the dispersion of clove essential oil through film. As a result, it showed an important antimicrobial activity against *Bacillus cereus, E. coli*, *Salmonella enteritidis*, and *S. aureus*, microorganisms that cause foodborne diseases. In addition, the presence of micro-fibrillated cellulose reinforced the protein matrix, increasing the mechanical strength and Young’s modulus of the films and improving their barrier properties to water vapor and oxygen [192].

The plant-based proteins have functional properties to form a diversified range of carrier systems with the ability to protect lipophilic bioactive compounds, control their release, and allow their delivery to a target site. However, it is notable that there are plant proteins with specific properties that are more indicated to form a kind of carrier structure due to their amino acid composition and structure. In addition, the type of plant protein, the interaction of these proteins with other materials (such as proteins, carbohydrates), and the production methods and processing conditions used can influence the characteristics of the carrier matrix and its affinity with the bioactive compounds. Therefore, the construction of a carrier structure requires a deep analysis of multiple factors that can affect its specificities and that determine its capacity to protect the bioactive compounds against external factors and its behavior through the gastrointestinal tract, defining its capacity to act as a delivery platform.

## 5. Can Lipophilic Bioactive Compounds Influence the Structure/Functionality of the Plant Protein-Based Delivery Systems?

**Figure 4:**
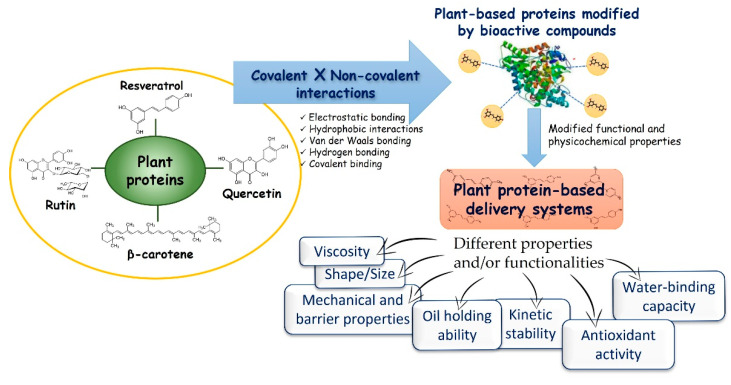
Interactions between proteins and lipophilic bioactive compounds and their effects on plant-based protein structure and characteristics of plant protein-based carrier platforms.

Bioactive compounds may contribute to the dynamics and molecular structure of delivery systems. They may interact chemically and/or physically with the protein matrix, inducing desirable or undesirable changes in the protein-based carriers. Proteins and lipophilic bioactive compounds, such as polyphenols (flavonoids and phenolic acids) and carotenoids, can form complexes via non-covalent physical interactions (e.g., electrostatic, hydrophobic, van der Waals, and hydrogen bonding) and/or conjugates through covalent bonds [193]. Non-covalent interactions, due to their unstable nature, are reversible and relatively weaker than covalent counterparts; however, both interactions can promote multiple changes in the protein structure [194] and, therefore, in the characteristics/functionalities of matrices built from plant proteins after interaction with lipophilic bioactive compounds (Figure 4).

For instance, soy protein–carvacrol interactions in films resulted in some transformation of the protein’s secondary structure from β-sheet conformation to α-helix [190]. Differently, the zein–quercetagetin conjugation reduced the content of the α-helix and β-sheet and increased the content of β-turns and random coils [195]. Binding with lipophilic polyphenols can also modify the physicochemical and functional properties of native proteins, such as surface hydrophobicity, thermal stability, wettability, solubility, surface activity, and antioxidant/antimicrobial activities. For example, a recent study reported that the interaction with quercetin-rich onion skin extracts decreased the solubility of the lentil protein and negatively affected its foaming and emulsifying properties [196]. On the other hand, the interfacial tension of native soy protein at the soybean oil/phosphate buffer interface decreased with increasing rutin concentration [197]. Similarly, the binding with curcumin enhanced the surface activity of the walnut protein, which quickly adsorbed at the air/water interface, further reducing surface tension [144]. Furthermore, curcumin promoted changes in the pea protein structure that resulted in reducing its surface hydrophobicity [140]. However, the presence of resveratrol had no impact on zein wettability [109].

With regard to antioxidant activity, the ability of zein to scavenge DPPH and ABTS radicals improved after its non-covalent and covalent binding to quercetagetin. However, no change in the thermal stability of zein was observed [195]. In addition to protein properties, the interaction of protein–polyphenol can also alter the characteristics and functionalities of the bioactive phenolics. For instance, curcumin’s aqueous solubility and stability increased after its complexation with pea protein isolate. Under physiological pH at 28 °C, about 72% of free curcumin was degraded after 30 min and 82% within 4 h, while the complexes were stable and retained 96% of curcumin after 4 h [145]. A similar improvement in curcumin solubility was achieved after its complexation with walnut protein [139]. Notably, the extent and range of these changes depend on the bond nature, reaction conditions (temperature, pH), type of protein (composition and sequence of amino acids, structural conformation), and phenolic compound (number of hydroxyl groups and benzene rings, molecular size, polarity) [198,199].

Proteins and lipophilic bioactive compounds have their own attributes; however, the new molecule formed by their interaction has distinct characteristics. Thus, functional compound-modified plant protein-based delivery systems may have different properties and/or functionalities than those built from native protein, such as (i) self-assembly capacity, shape and size particles in self-assembled systems; (ii) mechanical and barrier properties in films; (iii) interfacial layer properties, droplet size, rheological properties, kinetic and oxidative stability in emulsified/foam systems; and, (iv) viscosity, water/oil-holding capacity, stability, and rheological properties in gelled systems [193]. Indeed, the addition of resveratrol increased the Z-average diameters of the pea protein-based nanocomplexes and nanoparticles [146]. In addition to affecting the size, the interaction with quercetagetin and the nature of the protein–phenol bond altered the morphology of zein nanoparticles. Compared with highly deformed native zein nanoparticles, the zein–quercetagetin complexes formed larger spheres with a slightly irregular shape, while the conjugates produced smaller and smooth-surfaced particles [195].

In soy protein-based films, emulsified carvacrol reduced the strength and rigidity of the films and increased their extensibility, although no effect on water vapor permeability was observed [190]. Otherwise, the addition of rutin improved the mechanical properties of soy protein-based films, and they were stronger but less flexible than those without this phenolic. Rutin also had a desirable effect on reducing water vapor permeability from 2.3 g mm/m^2^ h kPa to 1.2 g mm/m^2^ h kPa, which was attributed to an increase in matrix compactness caused by protein–rutin crosslinking [200]. In emulsified systems, rice bran protein-stabilized nanoemulsions (3% *w/v* protein at pH 9.0) showed larger droplets after incorporating an increased quercetin content [172]. On the other hand, a gradual reduction in the droplet diameter and an improvement in the physical stability of the soy protein-emulsions were observed with increasing rutin concentration (0–0.2 mg/mL). The rutin displaced a significant content of initial interfacial protein and competitively adsorbed at the interface, forming mixed interfacial layers [197]. Likewise, the interactions with rutin appreciably increased the foaming properties of soybean proteins. At pH 7.0, the foaming capacity and foaming stability of the rutin–soybean protein complexes (28.33% and 14.22%, respectively) were significantly higher when compared with those of soybean protein alone (19.64% and 32.95%, respectively), which was attributed to decreased gas bubble size and the formation of a thicker and more rigid interfacial layer [201]. The interactions between zein nanoparticles and cinnamon essential oil also changed the interfacial layer characteristics, significantly increasing storage modulus (G′) values in Pickering emulsions [202].

The reaction between polyphenols and proteins can also positively affect the oxidative stability of carrier systems containing lipids. For example, the soy protein–resveratrol complexes stabilized corn oil/water emulsions and showed increased oxidative stability with reduced lipid hydroperoxides and volatile hexanal [203]. In more complex systems, the incorporation of β-carotene within the dispersed and/or continuous phase affected the zein-based emulsion gel properties differently. β-carotene interacted with zein via hydrophobic forces at the interface and the continuous glycerol phase. Within the continuous phase, the β-carotene reduced hardness but enhanced the gels’ viscosity, oil-holding ability, and water-binding capacity. In contrast, within the oil phase, it increased the elastic properties and mechanical stability of gels [204].

As previously reported, interactions between plant proteins and lipophilic bioactive compounds can simultaneously modify both molecules’ characteristics and delivery systems’ properties built from their complexes/conjugates. Pre-existing intra-/inter-molecular interactions in native protein-based carrier systems are changed, and new molecular interactions are induced in modified plant protein-based delivery systems, which can cause changes in the structural organization of their matrix, interfering with their behavior throughout the digestive tract [75]. During the digestion process, the structural conformation and characteristics of the carrier systems can modulate their interaction with the molecules from simulated digestive fluids/enzymes, altering their gastrointestinal fate (protein digestibility) and bioaccessibility/bioavailability of bioactive compounds loaded in them [205,206,207,208].

## 6. Conclusions and Future Perspectives

Consumers are increasingly aware of the relationship between diet quality, promoting health and well-being, and environmental sustainability related to food production and processing. In this context, the demand for delivery systems for bioactive compounds and new ingredients as carrier materials grows. Plant-based proteins are becoming fast-growing and innovative carrier ingredients in the food, nutraceutical, and pharmaceutical industry due to their advantages over their animal-derived counterparts, especially concerning sustainability aspects and ethical implications. However, the native globular structure of plant-based proteins is frequently destroyed during the extraction and recovery processes, promoting their functionality. Furthermore, these proteins showed antinutritional factors and undesirable sensorial characteristics.

Approaches to modifying the properties of plant-based proteins have been used to improve their techno-functionality and address their limitations. Therefore, new extraction and purification technologies aiming to preserve the characteristics of native plant proteins are sought to meet market demand for functional, eco-friendly, and sustainable products. Furthermore, these novel technologies and modification approaches of plant-based proteins should meet the requirements of green chemistry, allowing the development of sustainable carrier materials with tailored properties. Furthermore, new sources of plant-based proteins need to be found, and, in this sense, agro-industrial waste and by-products (such as sesame bran and green biomass from Jackfruit (*Artocarpus heterophyllus* Lam)) are attractive alternatives, as they are convenient and sustainable raw materials and have high protein content with an excellent nutritional profile [209,210]. In addition, the functional properties and bioactivity of the plant-based proteins obtained from these new sources must be deeply studied.

Knowing different plant protein characteristics will allow the rational choice of a modification approach that can improve their nutritional, sensorial, and techno-functional properties, aiming at a specific application. In addition, this knowledge will allow the rational design of delivery systems with enhanced properties based on plant protein only, blends of plant proteins, or their hybrids with dairy and animal counterparts.

Regarding the project of delivery platforms, more sophisticated structures must be reached by the simultaneous use of different types of carrier materials, in the form of a simple combination (e.g., complexes, conjugates, fibrils) or the formation of more complex systems (e.g., Pickering emulsion stabilized by nanogels/conjugates/fibrils or emulsion/fibrils-filled gels and films). Such combinations also emerge as the future for developing delivery systems with improved stability and high absorption and better bioactive compound release in gastrointestinal conditions.

## Figures and Tables

**Table 1:** A summary of recent studies evaluating the effects of modification approaches on plant-based protein properties.

Modified Characteristics	Protein Type	Modification Approach	Mechanism	Reference
Increased solubility and water hydration ability	Pea protein	Microwave heating-assisted glycation—Maillard reaction (glucose, fructose, and allulose)	Heat and covalent bond between protein and polysaccharides	[82]
Reduced particle size, increased solubility, and improved thermal stability and gelling ability	Oat protein	Ultrasound treatment	Cavitation and heat	[83]
Improved water binding capacity, oil holding capacity, and emulsifying properties	Rice bran protein	Radio frequency treatment	Heat	[84]
Better water binding capacity and oil holding capacity, emulsifying and gelation properties	Pea protein	Acylation (acetylation and succinylation) and/or conjugation (guar gum)	Introduction of an acyl group to the protein and covalent bond between protein and polysaccharide	[85]
Improved gelling capacity	Soy proteins	Conventional heat treatment or ultrasound treatment	Cavitation and heat	[86]
Improved gelling capacity	Pea protein	pH-shifting method	pH (alkaline treatment)	[87]
Enhanced thermal stability of protein, strengthened formation of gels, and improved textural properties (hardness, gumminess, and chewiness)	Sweet potato protein	High hydrostatic pressure and/or microbial transglutaminase	Pressure and covalent crosslinking (ε-(γ-glutaminyl) lysine isopeptide bonds)	[88]
Better water absorption capacity, water solubility, thermal stability, gel strength, gelation capacity, and in-vitro digestibility	Soy protein	Ultrasound treatment	Cavitation and heat	[89]
Improved emulsifying properties	Kidney bean protein	Conventional heat treatment/high-pressure homogenization	Heat and pressure	[90]
Enhanced emulsifying activity	Soy protein	Heat treatment and/or glutaminase deamidation	Heat and transformation of amide groups of glutamine and asparagine residues into carboxyl groups	[91]
Increased solubility and improved emulsifying and foaming capacity	Zein	Hydrolysis-glycation by transglutaminase (chitosan)	Breakdown into smaller peptides enzymatically and covalent bond between protein and polysaccharides	[92]
Improved foaming and emulsifying properties	Rice glutelin protein	Amyloid fibrilization	Heat, acidic condition, hydrolysis, and aggregation	[93]
Improved emulsifying activity and in-vitro antioxidant activity	Oat protein	Enzymatic hydrolysis	Breakdown into smaller peptides enzymatically	[94]
Improved emulsifying and foaming properties, solubility in water, andreduced allergenicity	Soy protein	Cold atmospheric plasma	Temperature and pressure (combination of thermal, mechanical, nuclear, and electrical energy sources)	[95]
Enhanced antioxidant activity	Rice protein	Hydrolysis-assisted electron beam irradiation	Breakdown into smaller peptides enzymatically and ionizing irradiation	[96]
Reduced off-flavors andimproved taste	Pea protein	Fermentation	Reduction of pH (lactic acid) and removal of aldehyde and ketone with low alcohol production	[97]
Decreased bitterness and improved aroma impression	Lupin protein	Fermentation	Degradation of glucose and production of lactic acid (pH acidification)	[98]
Improved solubility	Pea protein	Protein–protein blend/low-temperature homogenization	Cold temperatures and sodium citrate addition (disruption of the micelle structure of casein), hydrophobic interactions (casein core and hydrophobic amino acids of pea proteins), and pressure	[99]

**Table 2:** Recent studies on encapsulation of lipophilic bioactive compounds using plant-based proteins as carrier material.

Carrier Material	Lipophilic Bioactive Compound	Colloidal Carriers	Encapsulation Efficiency	Bioaccessibility/ Bioavailability	Outcomes	Reference
Walnut protein	Curcumin	Complexes	60.7%	-	Encapsulation in walnut proteins improved water solubility of curcumin and promoted its sustained release under simulated gastrointestinal conditions.	[144]
Pea protein	Curcumin	Pea protein-curcumin complex	98.6%	72%	Complexed curcumin showed improved water solubility (1.02 mg/g), bioavailability, and storage stability under physiological conditions compared to the free curcumin.	[145]
Pea protein	Resveratrol	Nanoparticles Nanocomplexes	74.08%	-	Water solubility, chemical stability, and antioxidant capacity of resveratrol were improved in pea protein nanoparticles and nano complexes compared to free resveratrol.	[146]
Zein-chitosan	Resveratrol	Particles	91%	47%	Encapsulation of resveratrol in zein-chitosan particles decreased ABTS but increased the DPPH scavenging capacity. The chitosan coating improved the storage stability of resveratrol and allowed its sustained in-vitro release.	[147]
Soy protein -cellulose nanocrystal	Curcumin	Nanoparticles	88.3%	81%	Nanoparticles produced with soy protein isolate: cellulose ratio of 6:1 showed good stability under a wide range of salt, heat, and pH conditions. The soy protein-cellulose nanocrystal composite nanoparticles reduced the release of curcumin in the stomach and allowed its highly controlled release in the intestine.	[148]
Oat protein-*Pleurotus ostreatus* β-glucan	β-carotene	Emulsion stabilized by Maillard conjugates	-	16–36% (bioaccessibility) 2–11% (bioavailability)	Oat protein isolate—*Pleurotus ostreatus* β-glucan conjugates protected emulsion against environmental stresses, facilitated its complete lipidic digestion, and improved the bioaccessibility and oral bioavailability of β-carotene.	[149]
Pea protein/curcumin/pectin	β-carotene	Emulsion stabilized by curcumin-protein-polysaccharide complex	76.15%	-	The pea protein/curcumin/pectin complex improved the physical stability of emulsions and chemical stability of β-carotene when exposed to UV light (76.15%, 8 h) and/or heat treatment at 25 (91.50%) and 50 °C (74.35%) for 30 days.	[150]
Soy protein-alginate	Lycopene	Emulsion gel beads	97%	0–12%	Emulsion gel beads at pH 3.0 showed lower mechanical strength, higher storage stability, and higher bioaccessibility of lycopene than those produced at pH 7.0 and 5.0.	[151]
Zein	Curcumin/ β-carotene	Pickering emulsions stabilized by curcumin-zein nanoparticles	47–96% (curcumin) 73–97% (β-carotene)	5–75% (curcumin) 13–28% (β-carotene)	Co-encapsulation improved the chemical stability of β-carotene and curcumin synergistically. The higher particle concentration and heating temperature retarded the free fat acid release, with lower bioaccessibility of nutraceuticals. Conversely, the lower pressure (≤100 MPa) promoted lipolysis and enhanced the bioaccessibility of nutraceuticals.	[152]
Zein-pectin	Cinnamon essential oil	Pickering emulsion stabilized by zein-pectin composite nanoparticles	-	-	Zein-pectin-based Pickering emulsions showed good dispersibility and sustained-release ability, the cinnamon essential oil improving its antibacterial performance compared to pure essential oil.	[153]
Zein-pectin	Peppermint oil and resveratrol	Emulsion stabilized by resveratrol-loaded zein-pectin complex particles	88% (peppermint oil) 99% (resveratrol)	-	Emulsions stabilized by resveratrol-loaded zein-pectin complex particles showed improved antimicrobial activity, physical and chemical stability, and prolonged antimicrobial efficiency of peppermint oil and resveratrol against *Staphylococcus aureus* and *Salmonella Typhimurium*.	[109]
Soy protein-wheat bran arabinoxylan	β-carotene	Emulsion-filled gels	-	76%	The soy protein (SPI)-wheat bran arabinoxylan (WBAX) emulsion-filled gels showed superior strengths and stabilities to those of the SPI-WBAX hydrogels and the WBAX or SPI emulsion-filled gels. The SPI-WBAX emulsion-filled gels improved the sustained release of β-carotene during digestion compared to the WBAX emulsion and SPI emulsion-filled gels.	[154]
Soy protein	Vitamin D_3_	Emulsion-filled gels	103–152 μg of vitamin D_3_/g gel	-	The application of mechanical stirring (800 rpm; 10–30 min) increased the solubility and decreased the particle size of soy protein (11–15%), affecting the microstructure and rheological properties of the heat-set gels. The gels of soy protein filled with Brazil nut oil emulsion were effective in protecting vitamin D3, presenting good retention over 30 days of storage (around 75% for gel produced with 15% of protein pretreated at 800 rpm for 30 min).	[155]
Wheat gliadin	Quercetin	W/O/W emulsion-filled gels	97.2%	-	The emulsion gels improved the quercetin solubility under simulated gastrointestinal conditions, which led to a four-fold increase in their effective bioaccessibility.	[156]
Zein-chitosan	Cinnamal-dehyde	Chitosan/zein-cinnamaldehyde nano-cellulose composite film	-	-	The addition of cinnamaldehyde increased water resistance of the film and contributed to a more flexible and dense film structure. Furthermore, coating with the chitosan/zein-cinnamaldehyde nano-cellulose composite film delayed yellowing and maintained the quality of mango during storage at ambient temperature, and the respiration rate and weight loss of mangoes were significantly inhibited.	[157]
Zein-chitosan	Oregano essential oil	Emulsion-based active films	-	-	The control film, composed of zein chitosan and oregano essential oil, presented good antimicrobial and antioxidant activity. However, the addition of phenolics (tea polyphenols, propolis flavones or grape proanthocyanidins) increased inhibition zone for *E. coli* and *Bacillus subtilis*, and these films had considerable potential for extending the shelf-life of fresh pork by delaying spoilage.	[158]
Soy protein- cellulose nanocrystals	Curcumin	pH-responsive films	-	-	The nanocomposite films were responsible for delaying the release of curcumin from the film matrix. The film composed of cellulose nanocrystals and curcumin nanocapsules displayed higher antiradical scavenging activity than that with free curcumin. Moreover, cellulose nanocrystals/curcumin nanocapsules film decreased the total volatile basic nitrogen of stored shrimp and visually monitored shrimp freshness in real-time.	[159]
